# Non-overdose acute care hospitalizations for opioid use disorder among commercially-insured adults: a retrospective cohort study

**DOI:** 10.1186/s13722-023-00396-9

**Published:** 2023-07-11

**Authors:** Sudha R. Raman, Cassie B. Ford, Bradley G. Hammill, Amy G. Clark, Dana C. Clifton, George L. Jackson

**Affiliations:** 1 Department of Population Health Sciences, Duke University School of Medicine, 215 Morris Street, Suite 210, Durham, NC 27701 USA; 2 Division of General Internal Medicine, Department of Medicine, Duke University School of Medicine, 2301 Erwin Road, Durham, NC 27710 USA; 3 Department of Pediatrics, Duke University School of Medicine, 2301 Erwin Road, Durham, NC 27710 USA; 4 Center of Innovation to Accelerate Discovery and Practice Transformation (ADAPT), Durham VA Medical Center, Durham Veterans Affairs (VA) Health Care System, 508 Fulton Street, Durham, NC 27705 USA; 5 Department of Family Medicine and Community Health, Duke University School of Medicine, 2100 Erwin Road, Durham, NC 27705 USA; 6 Peter O’Donnell Jr. School of Public Health, University of Texas Southweatern Medical Center, 5323 Harry Hines Blvd, Dallas, TX 75390 USA

**Keywords:** Opioid use disorder, Hospitalization, Buprenorphine, Health services research

## Abstract

**Background:**

Acute care inpatient admissions outside of psychiatric facilities have been increasingly identified as a critical touchpoint for opioid use disorder (OUD) treatment. We sought to describe non-opioid overdose hospitalizations with documented OUD and examine receipt of post-discharge outpatient buprenorphine.

**Methods:**

We examined acute care hospitalizations with an OUD diagnosis in any position within US commercially-insured adults age 18–64 years (IBM MarketScan claims, 2013–2017), excluding opioid overdose diagnoses. We included individuals with ≥ 6 months of continuous enrollment prior to the index hospitalization and ≥ 10 days following discharge. We described demographic and hospitalization characteristics, including outpatient buprenorphine receipt within 10 days of discharge.

**Results:**

Most (87%) hospitalizations with documented OUD did not include opioid overdose. Of 56,717 hospitalizations (49,959 individuals), 56.8% had a primary diagnosis other than OUD, 37.0% had documentation of an alcohol-related diagnosis code, and 5.8% end in a self-directed discharge. Where opioid use disorder was not the primary diagnosis, 36.5% were due to other substance use disorders, and 23.1% were due to psychiatric disorders. Of all non-overdose hospitalizations who had prescription medication insurance coverage *and* who were discharged to an outpatient setting (n = 49, 237), 8.8% filled an outpatient buprenorphine prescription within 10 days of discharge.

**Conclusions:**

Non-overdose OUD hospitalizations often occur with substance use disorders and psychiatric disorders, and very few are followed by timely outpatient buprenorphine. Addressing the OUD treatment gap during hospitalization may include implementing medication for OUD for inpatients with a broad range of diagnoses.

**Supplementary Information:**

The online version contains supplementary material available at 10.1186/s13722-023-00396-9.

## Background

Drug overdose is a leading cause of death for adults ages 18–45 years in the United States, including 80 816 opioid overdose deaths in 2021 [1]. Opioid-related hospitalizations, defined as hospitalizations for opioid overdose or opioid use disorder (OUD), have risen over the last decade. Despite strong evidence that medications for OUD are cost-effective and can reduce opioid-related overdose, all-cause mortality, and illicit drug use [2–4], less than 20% of the 2.1 million Americans with OUD receive any type of treatment each year.[5].

Acute care inpatient admission outside of psychiatric facilities have been increasingly identified as a critical touchpoint for OUD treatment [6], recognizing that opioid-related hospitalizations have high rates of readmissions [7–9] and death in the 30 days after discharge [10–12]. Most hospitalized patients with documentation of overdose or OUD in the hospitalization record do not receive treatment while in hospital [13], and a large proportion (40%) do not receive treatment within 30 days of discharge [14]. Among hospitalizations for conditions that are likely consequent to or exacerbated by OUD, such as endocarditis [10, 15], cellulitis [16], and osteomyelitis [17], discharges ‘against medical advice’ are common (18) and medication for OUD following discharge is infrequent [19].

Prior descriptions about OUD treatment during and after hospitalizations do not differentiate between OUD-related hospitalizations with and without overdose. While hospitalization is recognized as an opportunity for OUD treatment, we are interested to understand more about hospitalizations in a general medical setting without overdose—where OUD is either the acute reason for hospitalization or significant enough for OUD to be documented for billing purposes. As even an acute event such as a hospital-attended overdose is unlikely to precipitate medication for OUD [4], efforts continue to understand touchpoints within general medical settings as reachable moments. Accordingly, we chose to focus on OUD hospitalizations without overdose as a distinct encounter that may represent an important intervention point. The importance of all potential treatment opportunities has been highlighted by the recent increase in OUD prevalence during the COVID-19 pandemic [20, 21].

Commercially-insured populations, who account for about 17% of opioid-related hospitalizations [22] and may have fewer financial barriers to access to post-discharge OUD care than other insured groups, are an unique population to assess the extent of OUD treatment after discharge from acute care settings. Ongoing clinical trial and observational evidence is building for the effectiveness of OUD treatment initiation for medical inpatients, primarily in the form of addiction consult services, medication, and linkage to follow-up services [23–25]. To delineate the scope and target of potential interventions by hospitalists to identify and treat OUD appropriately in the inpatient setting, we require a more complete understanding of the full range of hospitalizations where OUD is present. The objective of this research is to describe non-opioid overdose hospitalizations with documented OUD. We leverage very large, real-world longitudinal commercial claims data to capture both hospitalization characteristics (e.g. high-frequency diagnoses, discharge status) and the frequency of post-discharge outpatient OUD medication treatment.

## Methods

### Study population

This study used data from the IBM MarketScan Commercial Claims and Encounters Database for the years 2013–2017. The MarketScan database captures individual-level data about all billed insurance claims for outpatient and inpatient services, and prescription drugs of over 40 million employees and their dependents covered by large employers and regional health plans in all 50 states [26]. These data also allow for the real-world examination of post-discharge treatment trajectories (including medication use) across settings and add to previous research that used hospitalization-only data without follow-up to the outpatient setting [13, 22], or described post-discharge treatment prior to the major changes due to the 2014 Affordable Care Act [14].

The study cohort included adults aged 18 to 64 years who were hospitalized in a non-psychiatric acute care hospital between July 1, 2013—December 31, 2017, with an ICD-9-CM/ICD-10-CM diagnosis code for “opioid use, abuse, or dependence”, hereafter termed opioid use disorder (OUD, see Additional file 1: Appendix Table 1). We considered OUD to be the primary reason for hospitalization if listed in the first diagnosis position; an OUD diagnosis in any other position was considered a secondary diagnosis.

We examined hospitalizations with documented OUD, and excluded those with diagnosis codes indicating OUD in remission (Additional file 1: Appendix Table 2). To capture a distinct hospitalization (a potential initial opportunity for treatment), and avoid including readmissions within 6 months (which may be unique in terms of complexity, or indication for OUD treatment), we included hospitalizations preceded by at least 6 months of continuous enrollment, without prior OUD hospitalizations in that period. To ensure appropriate follow-up, at least 10 days of medical insurance enrollment following discharge was required. An individual could have more than one hospitalization included in the analyses as long as each hospitalization met the inclusion criteria (i.e., at least 6 months without a prior OUD-related hospitalization). We focused on hospitalizations without evidence of opioid overdose, defined by ICD-9 and ICD-10 codes for opioid poisoning or adverse effects (Additional file 1: Appendix Table 3). Opioid overdose hospitalizations were quantified and then excluded from further analysis.

### Measures

Post-hospitalization medication for OUD was defined as an outpatient prescription fill claim for buprenorphine or buprenorphine/naloxone (Additional file 1: Appendix Table 4) within 10 days of hospital discharge. Three medications are FDA-approved for OUD (methadone, naltrexone, and buprenorphine); methadone is only administered at highly regulated clinics (not consistently included in these data), and naltrexone is infrequently used as it requires a period of abstinence prior to initiation [27]. We focused on outpatient medication because of its importance to treatment continuity in the community setting. Buprenorphine formulations are the most widely used prescription OUD medication for commercially-insured individuals in the outpatient setting [28]. In these data, we cannot observe inpatient medications for OUD or methadone administration, therefore aimed to assess medication continuity by observing the presence of prescriptions for buprenorphine filled in the outpatient setting during the 10-day post-discharge period. The 10-day period was chosen to reflect clinical practice of prompt outpatient follow up after discharge [29], and early post-discharge prescriptions most likely to be related to hospital care. Not all commercial insurance policies cover outpatient medications, therefore post-hospitalization medication use for people without outpatient medication coverage cannot be observed in these data. For patients who had prescription medication insurance coverage and were discharged to an outpatient setting, we calculated the proportion of hospitalizations followed by prescription dispensing for buprenorphine.

To describe individuals, we used age group (18–25, 26–34, 35–44, 45–43, 55–64), gender (male/female), and the beneficiary’s relationship to the insured person (employee, spouse, child/other). For hospitalizations, we identified primary and secondary diagnoses (ICD9 and ICD-10 codes), and admission type (psychiatric/substance abuse, surgical, medical, maternity, newborn, unknown). We identified alcohol-related diagnoses using ICD code 303*-alcohol intoxication, 305*- alcohol abuse, 291*- alcohol induced conditions, and F10-alcohol related disorders. Due to the heterogeneity and number of individual codes for closely related conditions, we grouped similar diagnosis into larger categories; diagnosis-related groups (DRG, a method to categorize similar hospitalizations into mutually exclusive groups), and major diagnostic categories (MDC, groupings of DRGs). We also examined length of stay (days), discharge status (home, self-directed, transfer, other), the proportion of patients with prescription medication coverage. Though documented codes may indicate the presence or absence of ‘rehabilitation therapy’, there may be substantial variation by facility in the use of these specific codes. Therefore, we focus on the more concrete data about post-discharge treatment, and examine the proportion who filled outpatient buprenorphine prescriptions within 10 days of discharge. See Additional file 1: Appendix Table 7 for discharge codes that indicated an outpatient discharge status.

### Analysis

We described individual characteristics (age, gender, insurance type), in all hospitalizations, those that noted OUD as the primary diagnosis (Primary-OUD) and those that had OUD in any other position (Secondary-OUD). Among hospitalizations with documented OUD, and without overdose, we described primary and secondary diagnoses, diagnosis-related groups, major diagnostic categories, and, as a post hoc analysis, the proportion of all hospitalizations that included alcohol-related diagnoses. We described characteristics of the hospitalization: length of stay, discharge status (home, self-directed/AMA, other) and proportion of patients with prescription medication coverage. For those who had prescription medication coverage *and* who were discharged to an outpatient setting, we described the proportion who filled outpatient buprenorphine prescriptions within 10 days of discharge. Group differences (between Primary-OUD and Secondary-OUD) were evaluated using chi-square tests for categorical variables and Kruskal–Wallis tests for continuous variables. All analysis was conducted using SAS, Version 9.4 (SAS Institute). This study was determined exempt from review by the Duke Health Institutional Review Board (#00103945).

## Results

**Fig. 1:**
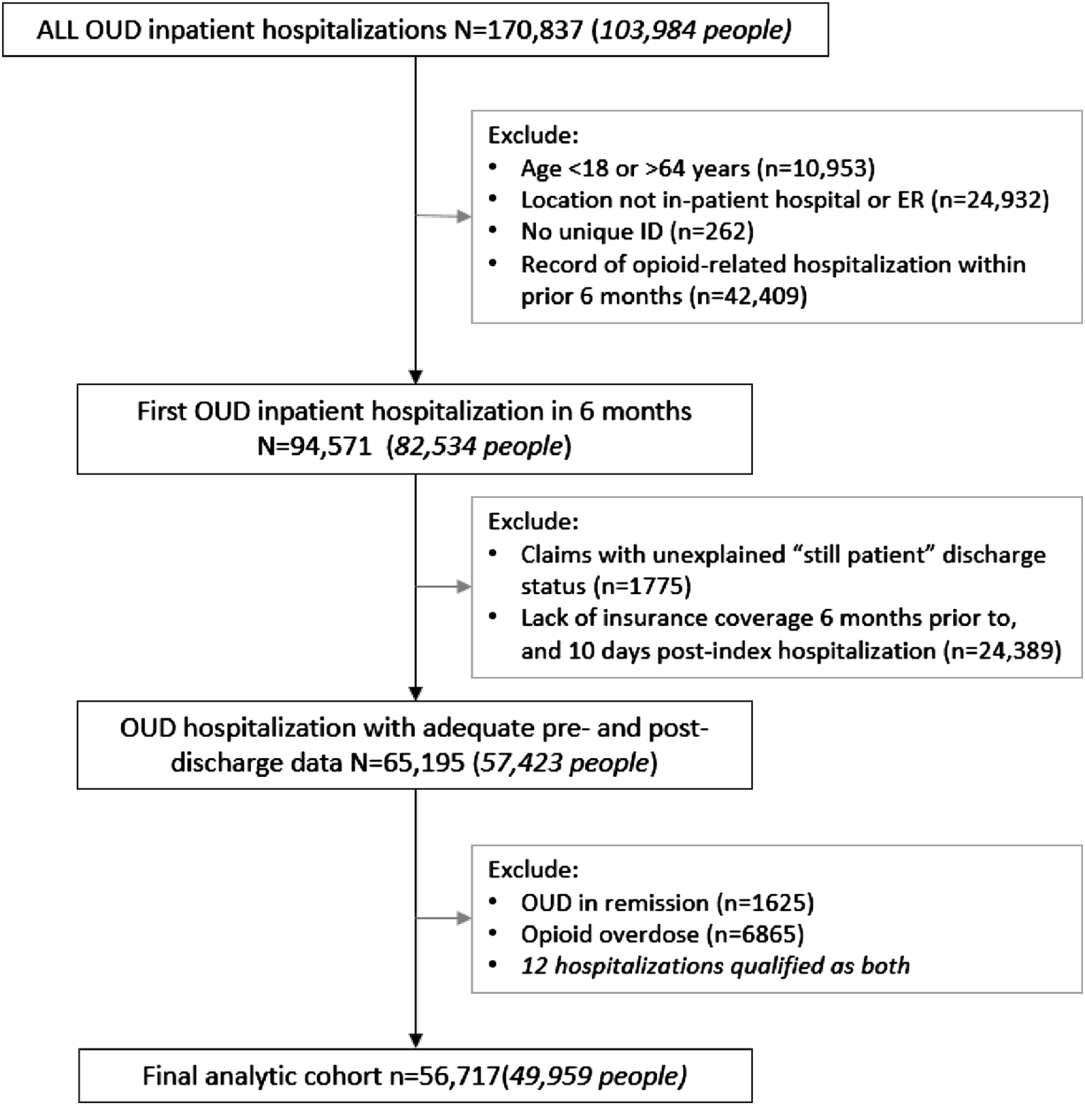
Study flow of hospitalizations. In cases where we identified multiple hospitalization claims for the same person during the same time period (overlapping or on day subsequent to the index visit), all with “still patient” as the discharge code, we excluded the claims

We identified 65,195 hospitalizations (57,423 individuals) with documentation of an OUD diagnosis between July 1, 2013 and December 31, 2017 after exclusions for patient age, missing ID, or patients without sufficient enrollment before or after a given hospitalization (Fig. 1). The majority of hospitalizations with documented OUD did not include opioid overdose; in fact, opioid overdose was noted in less than 13% of hospitalizations during each of the study years (Additional file 1: Appendix Table 5). Once overdose hospitalizations were excluded, the final study group included 56,717 hospitalizations amongst 49,959 unique individuals.

Of 56,717 hospitalizations with documented OUD without overdose, 43.2% were for a primary diagnosis of OUD (Primary-OUD) (Table 1). Of Primary-OUD hospitalizations, the majority (60.2%) were under age 26 and were more often males (66.1%) than females. In contrast, among the 56.8% of the hospitalizations with a secondary diagnosis of OUD (Secondary-OUD), individuals were more likely to be in older age groups (e.g. ages 45–54, 55–65 years old) and distributed more evenly by gender (p < 0.001).

**Table 1:** Characteristics of 56,717 hospitalizations with documented OUD without opioid overdose, by OUD diagnosis position (primary/secondary)

Variable	ALL	Primary-OUD	Secondary-OUD	p-value
N	56,717	24,495	32,222	
Age (years), Mean (SD)	34.38 (14.0)	29.8 (11.4)	37.9 (14.7)	< 0.001
Age Group (years)				< 0.001
18–25	26,095 (46.0%)	14,745 (60.2%)	11,350 (35.2%)	
26–34	7477 (13.2%)	3446 (14.1%)	4031 (12.5%)	
35–44	7519 (13.3%)	2732 (11.2%)	4787 (14.9%)	
45–54	7972 (14.1%)	2126 (8.7%)	5846 (18.1%)	
55–64	7654 (13.5%)	1446 (5.9%)	6208 (19.3%)	
Gender, Male	33,374 (58.8%)	16,201 (66.1%)	17,173 (53.3%)	<0 .001
Relationship to Insured				< 0.001
Employee	17,924 (31.6%)	6274 (25.6%)	11,650 (36.2%)	
Spouse	13,028 (23.0%)	3715 (15.2%)	9313 (28.9%)	
Child/Other	25,765 (45.4%)	14,506 (59.2%)	11,259 (34.9%)	
Admission Type				< 0.001
Psychiatric and Substance Abuse	3546 (6.3%)	24,310 (99.2%)	19,049 (59.1%)	
Surgical	8,300 (14.6%)	46 (0.2%)	3500 (10.9%)	
Medical	1,312 (2.3%)	0 (0.0%)	8300 (25.8%)	
Maternity and Newborn	43,359 (76.4%)	0 (0.0%)	1312 (4.1%)	
Unknown	200 (0.4%)	139 (0.6%)	61 (0.2%)	

^a^Group differences were evaluated using chi-square tests for categorical variables and Kruskal–Wallis tests for continuous variables

A large proportion (59.2%) of those with Primary-OUD hospitalizations were children of the insured and the admission type for almost all hospitalizations for this group were for ‘psychiatric and substance abuse’ (99.2%). For those who had Secondary-OUD hospitalizations, the majority (59.1%) were classified as ‘psychiatric and substance abuse’, one fourth (25.8%) were classified as ‘medical’ hospitalizations and a small proportion of secondary-OUD hospitalizations were classified as ‘surgical’ (10.9%) and ‘maternity and newborn’ (4.1%) (Table 1, p < 0.001).

Major Diagnostic Category (MDC) and contributing Diagnosis-Related Groups (DRG) categorizations showed a similar distribution. Among Primary-OUD hospitalizations, all were categorized as due to Alcohol/Drug Use or Induced Mental Disorders (MDC 20). Specifically, the majority were classified as three types of “alcohol, drug abuse or dependence”; 1) “without rehabilitation therapy without major complication or comorbidity” (88.5%), 2) “with rehabilitation therapy’ (5.1%) or 3) ‘left against medical advice’ (4.7%) (Table 2).

**Table 2:** Major Diagnostic Category (MDC) and contributing Diagnosis-Related Groups (DRGs, > 1%) of hospitalizations with documented OUD without opioid overdose, by OUD diagnosis position (primary/secondary)

%	Primary-OUD	%	Secondary-OUD
100	Alcohol/Drug Use or Induced Mental Disorders (MDC 20) Alcohol, Drug Abuse, or Dependence • 88.5% DRG 897: Without Rehabilitation Therapy Without MCC^a^ • 5.1% DRG 895: With Rehabilitation Therapy • 4.7% DRG 894: Left AMA^b^	36.5 23.1	Alcohol, Drug Abuse or Induced Mental Disorders (MDC 20) • 31.4% DRG 897: Alcohol, Drug Abuse or Dependence Without Rehabilitation Therapy Without MCC • 2.2% DRG 895: Alcohol, Drug Abuse or Dependence with Rehabilitation Therapy • 1.7% DRG 894: Alcohol, Drug Abuse or Dependence, Left AMA Mental Diseases and Disorders (MDC 19) • 18.4% Psychoses (DRG 885) • 3.2% Neuroses (DRG 881)
		6.7	Musculoskeletal System and Connective Tissue (MDC 8) • 1.6% DRG 470: Major Hip and Knee Joint Replacement or Reattachment of Lower Extremity Without MCC
		5.3	Digestive System (MDC 6) • 1.8% DRG 392: Esophagitis, Gastroenteritis and Misc Digestive Disorders Without MCC
		4.1	Pregnancy, Childbirth and Puerperium (MDC 14) • 1.5% DRG 775: Vaginal Delivery Without Complicating Diagnoses
		3.6	Respiratory System (MDC 4)
		3.5	Nervous System (MDC 1)
		2.8	Infectious and Parasitic DDs (MDC 18) • 1.3% DRG 871: Septicemia or Severe Sepsis without mechanical ventilation > 96 h with MCC
		2.7	Circulatory System (MDC 5)
		2.5	Skin, Subcutaneous Tissue and Breast (MDC 9) • 1.6% DRG 603: Cellulitis without MCC

^a^MCC—major complication or comorbidity

^b^AMA—against medical advice, referred to in the text as self-directed discharges

Over a third, 36.5%, of Secondary-OUD hospitalizations were also classified as Alcohol/Drug Use Disorders (MDC 20), 23.1% were categorized as psychiatric disorders (Mental Diseases and Disorders MDC 19, e.g. ‘psychoses’, 18.4%; and ‘depressive neuroses’, 3.2%). The use of MDCs enabled the grouping of the remaining very heterogeneous DRGs into common body systems. Notably, 6.7% of Secondary-OUD hospitalizations were classified as musculoskeletal system/connective tissue-related; 4.1, 2.8 and 2.5% were classified as due to pregnancy and childbirth-related, infectious and parasitic diseases and skin, and subcutaneous tissue-related, respectively (Table 2).

We examined alcohol-related diagnoses in the whole study group and found that of the 56,717 hospitalizations, 21,000 (37.0%) had documentation of an alcohol-related diagnosis codes. We also examined the primary ICD-9-CM and ICD-10-CM diagnoses among Secondary-OUD hospitalizations and found that dependence or withdrawal from alcohol were common. Diagnoses that may be injection drug use-related, such as endocarditis, cellulitis, and osteomyelitis were less common (e.g. Cellulitis of Right Upper Limb (1.3%) (Additional file 1: Appendix Table 6).

The average length of stay for Primary-OUD hospitalizations was 8.5 days, as compared to 5.8 days for Secondary-OUD hospitalizations (p < 0.001). The majority of all hospitalizations ended with a discharge to home or self-care (Primary-OUD 57.9%, Secondary-OUD 78.0%, p < 0.001). A small but notable proportion left ‘against medical advice’ (Overall, 5.8%, Primary-OUD, 4.1%, Secondary-OUD, 7.1%, p < 0.001). About 13% of each group did not have commercial insurance for medications during the post-discharge period (Overall 12.7%). Of those who had prescription medication coverage and who were discharged to an outpatient setting, a small proportion had evidence of outpatient prescription for buprenorphine or buprenorphine/naloxone within 10 days of discharge; this was similar between the two groups (Overall 8.8%, Primary-OUD 9.0%, Secondary-OUD 8.7%, p = 0.35) (Table 3).

**Table 3:** Hospitalization treatment characteristics by OUD diagnosis position (primary/secondary)

Variable	All	Primary-OUD	Secondary- OUD	p-value
N	56,717	24,495	32,222	
Length of Stay (days), Mean (SD)	7.0 (7.8)	8.5 (9.0)	5.8 (6.4)	< 0.001
Discharge Status (n, %)				<0 .001
Discharged to ‘home or self-care’, or ‘home health service’	39,330 (69.3)	14,188 (57.9)	25,142 (78.0)	
Self-directed discharge/AMA	3282 (5.8)	1006 (4.1)	2276 (7.1)	
Transfer to another facility^a^	2208 (3.9)	1162 (4.7)	1046 (3.2)	
Other	11,897 (21.0)	8139 (33.2)	3758 (11.7)	
Prescription medication coverage^b^ (n, %)	49,502 (87.3%)	21,752 (88.8%)	27,750 (86.1%)	< 0.001
Outpatient Prescription medication observable^c^ (n, %)	40,237 (70.9%)	15,848 (64.7%)	24,389 (75.7%)	< 0.001
Post-discharge medications (n, %^d^)				
Buprenorphine or buprenorphine/naloxone	3550 (8.8%)	1424 (9.0%)	2126 (8.7%)	.35

Primary-OUD: hospitalizations with a primary diagnosis of OUD; Secondary-OUD, hospitalizations with OUD in any position other than first. Group differences were evaluated using chi-square tests for categorical variables and Kruskal–Wallis tests for continuous variables

^a^Transfer to another facility includes all transfers to skilled nursing facilities and similar inpatient settings. ‘Other’ includes hospitalizations with missing discharge codes, where the discharge code indicated outpatient followup was not possible (e.g. deceased) or discharge location was not clearly home, transfer or self-directed discharge/AMA (e.g., ‘still patient’)

^b^Includes all hospitalizations for patients with prescription medication coverage for at least 10 days post-discharge

^c^Includes those who have prescription medication coverage *and* who were discharged to an outpatient setting (‘home or self-care’, or ‘home health service’, ‘left AMA’, etc.; see Additional file 1: Appendix Table 7 for full list)

^d^Percent of hospitalizations for individuals who have prescription medication coverage and were discharged to an outpatient setting

## Discussion

These results provide several novel insights about acute care hospitalizations with documented OUD in the commercially-insured population. First, opioid overdose accounts for only a minority of hospitalizations with documented OUD. Second, hospitalizations where OUD is not the primary diagnosis are often for other substance use disorders (such as alcohol-related conditions) or psychiatric disorders. Finally, very few non-overdose OUD hospitalizations are followed by timely outpatient OUD medications, and almost 6% end in a self-directed (discharge against medical advice, AMA), potentially exposing hospitalization as another treatment gap in the continuum of OUD care.

Results suggest that the impact of the opioid epidemic on acute care hospitals extends beyond admissions for overdose and OUD. Previous research has grouped all opioid-related hospitalizations together, often including both overdose and opioid use disorder and found that only a small proportion include treatment or rehabilitation [13, 22]. Our research adds to this literature by demonstrating that the majority of hospitalization with documented OUD are not for opioid overdose, and encompass a range of diagnosis groups that may indicate a widespread treatment opportunity among hospitalized patients with OUD.

Hospitalizations for OUD spanned a range of admission types, including psychiatric and substance use disorder diagnoses, medical, surgical, and maternity/newborn. Non-opioid substance use disorder diagnoses represented a substantial proportion of this group, highlighting the impact of polysubstance use in the opioid epidemic [30]. We also found psychiatric diagnoses as major contributors to hospitalizations with OUD, underscoring the overlap of mental health and substance use disorders [31] and the potential to treat this high-risk group [32]. With the understanding that not all hospitals have inpatient beds accredited for substance use disorder admissions, the implication of these findings is that a wide range of generalist clinicians may encounter hospitalized adults with OUD, not only those with addiction expertise.

Interventions to initiate OUD care in the inpatient setting initially focused on patients admitted with endocarditis related to injection drug use (including opioids) [33]. Increasingly, interventions to address OUD in medical settings are being championed by multidisciplinary teams in a broad range of hospitalized adults with OUD or other substance use disorders [25, 34]. Our results support broadening the target population of these inpatient interventions, with a particular focus on those with comorbid psychiatric or substance use disorders, such as alcohol use disorder. These results also suggest that medication for OUD initiated in the hospital setting will often need to occur in the context of other substance use disorders. Other substance use disorders can be a barrier to medication for OUD[35], and therefore interventions in this setting require that clinicians engage in training and support to provide buprenorphine for people with polysubstance use.

Our findings suggest challenges for OUD treatment during non-overdose OUD hospitalizations, as indicated by hospitalizations that require managing multiple other medical needs, short average length of stays and self-directed discharges. Shorter hospitalization length heightens the importance of timely screening, identification and treatment of OUD. Self-directed discharge (discharge against medical advice) in this population was threefold estimates of 1–2% in the general inpatient population [36]. Hospitalizations that end in a self-directed discharge increase risk for readmission, adverse events, or death due to incomplete treatment [37, 38]. Ascertainment and alleviation of the root causes of self-directed discharges will enable additional opportunity for OUD treatment and successful transitions to post-discharge community care.

Our results suggest that, similar to population-based estimates of OUD treatment in any setting [5] and substance use disorders in general [39], the majority of commercially-insured individuals hospitalized with OUD did not receive outpatient buprenorphine post-discharge. Though recent progress has been made to reduce administrative barriers (such as prior authorization) and increase access to medication for OUD [40], treatment after OUD-related emergency department or hospital encounters has been found to be low in both publicly-insured populations and commercially-insured populations [4, 41]. Our work demonstrates that these trends are similarly low for non-overdose hospitalizations.

Our result, that fewer than 10% of patients filled prescriptions for post-discharge outpatient buprenorphine within 10 days, is similar to results observed after opioid-related hospitalizations including overdose [42]. Few observed outpatient buprenorphine prescriptions might be partially explained by the transfer of patients to alternative facilities or patients who accessed methadone for OUD treatment (not captured in these data). However, overall, infrequent post-hospitalization outpatient treatment for OUD is important given that effective evidence-based OUD care requires medication continuity into community settings.

Translating this observed treatment gap into practice may include expanding care pathways and programs that better identify patients with OUD and assess the need for treatment during general acute care hospitalizations [43, 44], similar to the programs that actively screen and treat OUD in emergency or primary care settings [45, 46]. There is increasing evidence that establishing structures and increasing capacity among inpatient physicians to prescribe medication for OUD during all types of hospitalizations effectively decreases substance use disorder symptoms and drug use, avoids self-directed discharges, and engages people with OUD in post-discharge treatment [24, 25, 47, 48].

These results shed light on the gaps in care experienced by commercially-insured populations, who account for about 17% of OUD-related hospitalizations[22]. Commercial insurance barriers to access for medication for OUD, such as prior authorization, behavioral therapy requirements, and treatment limits are decreasing over time, but still may impede appropriate treatment of buprenorphine [49]. In addition to employed beneficiaries, these claims also describe young adults/dependents who may not have other insurance options or choose the better coverage of their parent’s plan. For example, we observed a large proportion of Primary-OUD hospitalizations for young adults who are dependent beneficiaries, highlighting the importance of recent allowances for parents to continue coverage of children up to 26 years of age, an age group highly affected by substance use disorders [50]. Additionally, we found that for about 13% of hospitalizations, individuals did not have outpatient medication insurance in the immediate post-discharge period so we were not able to observe outpatient medications for OUD for this group. There may be many explanations for this finding (e.g. medication coverage by another source), but indicate that lack of outpatient medication coverage may be a barrier to continuity of post-discharge outpatient medications for OUD.

These results should be considered in the context of the following limitations: We identified OUD using ICD-9 and ICD-10 codes that have not been validated, and may be used to reflect varying levels of OUD severity and indication for post-discharge treatment. In particular, the position of the OUD code may not be sufficient to indicate the severity of OUD or need for OUD treatment, especially when OUD is not in the primary diagnosis position. Misclassification (for example in the discharge status variable), may have affected our results. For example, if those in the ‘other’ discharge status category were actually discharged to an outpatient setting and less likely to be prescribed buprenorphine, our results about buprenorphine prescription would be underestimates. Though the proportion of OUD and overdose admissions did not change substantially over time, we cannot account for variation in increasing awareness and documentation of OUD, or that OUD may be more likely to be recorded during certain types of Secondary-OUD admissions (e.g. surgical or infection-related admissions). Claims data may not reflect all treatment received (e.g. other medication for OUD (such as methadone), counselling or residential treatment paid for by other insurance, self-pay, or federal and state programs). Additionally, medications prescribed during hospitalization or filled in the inpatient setting are not observable in these data. Medications recorded in claims data do not reflect written but unfilled prescriptions, and are a proxy for actual consumption. Lastly, these results reflect the national commercially-insured population with at least 6 months of continuous enrollment, an acknowledged minority of patients with OUD; and these results may not generalize to individuals who are commercially-insured for shorter periods (e.g. due to loss of employment due to substance use), publicly insured or uninsured or those who experience repeated hospitalizations within a 6 month period.

## Conclusions

This research illustrates that non-overdose acute care hospitalizations with documented OUD in general medical settings should be recognized as part of the continuum of OUD care provided by health systems. Our work adds to the evidence to support using resources to bolster both to initiate medications for OUD in the context of a broad range of hospitalization diagnoses and increase policies and practices that ensure continuity of mediation for OUD after hospital discharge.

## Supplementary Information

**Additional file 1: Appendix Table 1.** OUD code list. **Appendix Table 2.** OUD in remission code list. **Appendix Table 3.** Opioid overdose, poisoning, or adverse events code list. **Appendix Table 4.** Included OUD medications (as indicated in outpatient dispensed prescriptions. **Appendix Table 5.** Percent of hospitalizations with opioid overdose, by study year. **Appendix Table 6.** Most frequent primary diagnoses for Secondary-OUD* hospitalizations. **Appendix Table 7.** Discharge status codes used to indicate discharge to an outpatient setting.

## Data Availability

The datasets that support the findings of this study are available from IBM MarketScan but restrictions apply to the use and availability of these data, which were used under license for the current study, and so are not publicly available.

## Abbreviations

OUD: Opioid use disorder
ICD: International classification of diseases
FDA: U.S. Food and Drug Administration
DRG: Diagnosis-related group
MDC: Major diagnostic categories
AMA: Against medical advice

## References

[1] Ahmad FB, Cisewski JA, Rossen LM, Sutton P. Provisional drug overdose death counts. National Center for Health Statistics; 2023.

[2] Fairley M, Humphreys K, Joyce VR, Bounthavong M, Trafton J, Combs A (2021). Cost-effectiveness of treatments for opioid use disorder. JAMA Psychiatr.

[3] Sordo L, Barrio G, Bravo MJ, Indave BI, Degenhardt L, Wiessing L (2017). Mortality risk during and after opioid substitution treatment: systematic review and meta-analysis of cohort studies. BMJ.

[4] Larochelle MR, Bernson D, Land T, Stopka TJ, Wang N, Xuan Z (2018). Medication for opioid use disorder after nonfatal opioid overdose and association with mortality: a cohort study. Ann Intern Med.

[5] Wu LT, Zhu H, Swartz MS (2016). Treatment utilization among persons with opioid use disorder in the United States. Drug Alcohol Depend.

[6] Priest KC, McCarty D (2019). Role of the hospital in the 21st century opioid overdose epidemic: the addiction medicine consult service. J Addict Med.

[7] Reif S, Acevedo A, Garnick DW, Fullerton CA (2017). Reducing behavioral health inpatient readmissions for people with substance use disorders: do follow-up services matter?. Psychiatr Serv.

[8] Mark TL, Tomic KS, Kowlessar N, Chu BC, Vandivort-Warren R, Smith S (2013). Hospital readmission among medicaid patients with an index hospitalization for mental and/or substance use disorder. J Behav Health Serv Res.

[9] Peterson C, Liu Y, Xu L, Nataraj N, Zhang K, Mikosz CAUS (2019). National 90-day readmissions after opioid overdose discharge. Am J Prev Med.

[10] Leahey PA, LaSalvia MT, Rosenthal ES, Karchmer AW, Rowley CF (2019). High morbidity and mortality among patients with sentinel admission for injection drug use-related infective endocarditis. Open Forum Infect Dis.

[11] Song Z (2017). Mortality quadrupled among opioid-driven hospitalizations, notably within lower-income and disabled white populations. Health Affairs.

[12] Hser YI, Mooney LJ, Saxon AJ, Miotto K, Bell DS, Zhu Y (2017). High mortality among patients with opioid use disorder in a large healthcare system. J Addict Med.

[13] Peterson C, Xu L, Florence C, Mack KA (2019). Opioid-related US hospital discharges by type, 1993–2016. J Subst Abuse Treat.

[14] Ali MM, Mutter R. Patients who are privately insured receive limited follow-up services after opioid-related hospitalizations. The CBHSQ Report. Rockville (MD): Substance Abuse and Mental Health Services Administration (US); 2016. p. 1–4. 27054227

[15] Fleischauer AT, Ruhl L, Rhea S, Barnes E (2017). Hospitalizations for endocarditis and associated health care costs among persons with diagnosed drug dependence—North Carolina, 2010–2015. MMWR Morb Mortal Wkly Rep.

[16] Phillips KT, Anderson BJ, Herman DS, Liebschutz JM, Stein MD (2017). Risk factors associated with skin and soft tissue infections among hospitalized people who inject drugs. J Addict Med.

[17] Miller AC, Polgreen PM (2019). Many opportunities to record, diagnose, or treat injection drug-related infections are missed: a population-based cohort study of inpatient and emergency department settings. Clin Infect Dis.

[18] Schuller KA, Franz B, Cronin CE (2020). Patient characteristics affect discharge status for opioid-related infective endocarditis. Med Care.

[19] Kimmel SD, Walley AY, Li Y, Linas BP, Lodi S, Bernson D (2020). Association of treatment with medications for opioid use disorder with mortality after hospitalization for injection drug use-associated infective endocarditis. JAMA Netw Open.

[20] Volkow ND (2020). Collision of the COVID-19 and addiction epidemics. Ann Intern Med.

[21] Centers for Disease Control and Prevention. National Center for Health Statistics Monthly Provision Overdose Mortality. 2021.

[22] Peterson C, Xu L, Mikosz CA, Florence C, Mack KA (2018). US hospital discharges documenting patient opioid use disorder without opioid overdose or treatment services, 2011–2015. J Subst Abuse Treat.

[23] McNeely J, Troxel AB, Kunins HV, Shelley D, Lee JD, Walley A (2019). Study protocol for a pragmatic trial of the consult for addiction treatment and care in hospitals (CATCH) model for engaging patients in opioid use disorder treatment. Addict Sci Clin Pract.

[24] Englander H, Dobbertin K, Lind BK, Nicolaidis C, Graven P, Dorfman C (2019). Inpatient addiction medicine consultation and post-hospital substance use disorder treatment engagement: a propensity-matched analysis. J General Int Med.

[25] Wakeman SE, Metlay JP, Chang Y, Herman GE, Rigotti NA (2017). Inpatient addiction consultation for hospitalized patients increases post-discharge abstinence and reduces addiction severity. J Gen Intern Med.

[26] L.; H. IBM MarketScan Research Databases for life sciences researchers. Truven Health Analytics; 2018.

[27] Administration SAaMHS. Medications for Opioid Use Disorder. 2020. Contract No.: PEP20–02–01–006.

[28] Shah A, Duncan M, Atreja N, Tai KS, Gore M (2018). Healthcare utilization and costs associated with treatment for opioid dependence. J Med Econ.

[29] Trowbridge P, Weinstein ZM, Kerensky T, Roy P, Regan D, Samet JH (2017). Addiction consultation services—linking hospitalized patients to outpatient addiction treatment. J Subst Abuse Treat.

[30] Cicero TJ, Ellis MS, Kasper ZA (2020). Polysubstance use: a broader understanding of substance use during the opioid crisis. Am J Public Health.

[31] Jones CM, McCance-Katz EF (2019). Co-occurring substance use and mental disorders among adults with opioid use disorder. Drug Alcohol Depend.

[32] Robertson AG, Easter MM, Lin HJ, Frisman LK, Swanson JW, Swartz MS (2018). Associations between pharmacotherapy for opioid dependence and clinical and criminal justice outcomes among adults with co-occurring serious mental illness. J Subst Abuse Treat.

[33] Suzuki J (2016). Medication-assisted treatment for hospitalized patients with intravenous-drug-use related infective endocarditis. Am J Addict.

[34] Englander H, Weimer M, Solotaroff R, Nicolaidis C, Chan B, Velez C (2017). Planning and designing the improving addiction care team (IMPACT) for hospitalized adults with substance use disorder. J Hosp Med.

[35] Lin LA, Bohnert ASB, Blow FC, Gordon AJ, Ignacio RV, Kim HM (2021). Polysubstance use and association with opioid use disorder treatment in the US veterans health administration. Addiction.

[36] Kumar N (2019). Burden of 30-day readmissions associated with discharge against medical advice among inpatients in the United States. Am J Med.

[37] Choi M, Kim H, Qian H, Palepu A (2011). Readmission rates of patients discharged against medical advice: a matched cohort study. PLoS ONE.

[38] Eaton EF, Westfall AO, McClesky B, Paddock CS, Lane PS, Cropsey KL (2020). In-hospital illicit drug use and patient-directed discharge: barriers to care for patients with injection-related infections. Open forum infectious diseases..

[39] Lipari RNVH, S.L. Trends in substance use disorders among adults aged 18 or older. Rockville, MD: Substance abuse and mental health services administration; 2017. 28792721

[40] Ashford RD, Brown AM, McDaniel J, Neasbitt J, Sobora C, Riley R (2019). Responding to the opioid and overdose crisis with innovative services: the recovery community center office-based opioid treatment (RCC-OBOT) model. Addict Behav.

[41] Larochelle MR, Liebschutz JM, Zhang F, Ross-Degnan D, Wharam JF (2016). Opioid prescribing after nonfatal overdose and association with repeated overdose. Ann Intern Med.

[42] Naeger S, Ali MM, Mutter R, Mark TL, Hughey L (2016). Prescriptions Filled Following an Opioid-Related Hospitalization. Psychiatr Serv.

[43] Clifton D, Ivey N, Poley S, O'Regan A, Raman SR, Frascino N (2022). Implementation of a comprehensive hospitalist-led initiative to improve care for patients with opioid use disorder. J Hosp Med.

[44] Christian N, Bottner R, Baysinger A, Boulton A, Walker B, Valencia V (2021). Hospital buprenorphine program for opioid use disorder is associated with increased inpatient and outpatient addiction treatment. J Hosp Med.

[45] D'Onofrio G, Edelman EJ, Hawk KF, Pantalon MV, Chawarski MC, Owens PH (2019). Implementation facilitation to promote emergency department-initiated buprenorphine for opioid use disorder: protocol for a hybrid type III effectiveness-implementation study (project ED HEALTH). Implement Sci IS.

[46] Hunter SB, Ober AJ, McCullough CM, Storholm ED, Iyiewuare PO, Pham C (2018). Sustaining alcohol and opioid use disorder treatment in primary care: a mixed methods study. Implement Sci IS.

[47] Thompson HM, Faig W, VanKim NA, Sharma B, Afshar M, Karnik NS (2020). Differences in length of stay and discharge destination among patients with substance use disorders: The effect of Substance Use Intervention Team (SUIT) consultation service. PLoS ONE.

[48] Gryczynski J, Nordeck CD, Welsh C, Mitchell SG, O'Grady KE, Schwartz RP (2021). Preventing hospital readmission for patients with comorbid substance use disorder : a randomized trial. Annal Int Med.

[49] Huskamp HA, Riedel LE, Barry CL, Busch AB (2018). Coverage of medications that treat opioid use disorder and opioids for pain management in marketplace plans, 2017. Med Care.

[50] Merikangas KR, McClair VL (2012). Epidemiology of substance use disorders. Hum Genet.

