# Supplementary material for: Non-overdose acute care hospitalizations for opioid use disorder among commercially-insured adults: a retrospective cohort study
Source: Addict Sci Clin Pract. 2023 Jul 11;18:42. doi: 10.1186/s13722-023-00396-9 (PMC10337199; doi:10.1186/s13722-023-00396-9)
Supplement: Supplementary file 1 — Additional file 1: Appendix Table 1. OUD code list. Appendix Table 2. OUD in remission code list. Appendix Table 3. Opioid overdose, poisoning, or adverse events code list. Appendix Table 4. Included OUD medications (as indicated in outpatient dispensed prescriptions. Appendix Table 5. Percent of hospitalizations with opioid overdose, by study year. Appendix Table 6. Most frequent primary diagnoses for Secondary-OUD* hospitalizations. Appendix Table 7. Discharge status codes used to indicate discharge to an outpatient setting. [file 13722_2023_396_MOESM1_ESM.docx]

Non-overdose acute care hospitalizations for opioid use disorder among commercially-insured adults: a retrospective cohort study

Additional file

Appendix Table 1. OUD code list.

| **Diagnosis** | **ICD-9-CM** | **ICD-10-CM** |
| --- | --- | --- |
| ***Include*** | | |
| Opioid use disorder (OUD) | 304.00, 304.01, 304.02, 304.70, 304.71, 304.72, 305.50, 305.51, 305.52 | F11.10, , F11.120, F11.129, F11.20, , F11.220, F11.221, F11.222, F11.229, F11.23, F11.24, F11.250, F11.251, F11.259, F11.281, F11.282, F11.288, F11.29, |

Appendix Table 2. OUD in remission code list.

| **Diagnosis** | **ICD-9-CM** | **ICD-10-CM** |
| --- | --- | --- |
| Opioid use disorder, in remission | 304.03, 304.73, 305.53 | F11.11, F11.21 |

Appendix Table 3. Opioid overdose, poisoning, or adverse events code list.

| **Diagnosis** | **ICD-9-CM** | **ICD-10-CM** |
| --- | --- | --- |
| Opioid overdose (Unintentional, intentional, assault, or unknown) Includes methadone, heroine and other opiates | 965.00, 965.01, 965.02, 965.09, E850.0, E850.1, E850.2, E850.8 | T40.0X1A, T40.0X2A, T40.0X3A, T40.0X4A, T40.1X1A, T40.1X2A, T40.1X3A, T40.1X4A,  T40.2X1A, T40.2X2A, T40.2X3A, T40.2X4A, T40.3X1A, T40.3X2A, T40.3X3A, T40.3X4A, |
| Overdose by unspecified narcotics |  | T40.6X1A, T40.6X2A, T40.6X3A, T40.6X4A, T40.691A |
| Overdose by other synthetic narcotics |  | T40.4X1A, T40.4X2A, T40.4X3A, T40.4X4A, |

Appendix Table 4. Included OUD medications (as indicated in outpatient dispensed prescriptions)

| Buprenorphine |
| --- |
| Buprenorphine Hydrochloride |
| Buprenorphine/Naloxone |

Appendix Table 5. Percent of hospitalizations with opioid overdose, by study year.

| **Year** | **Hospitalizations (n)** | **Proportion with opioid overdose (%)** |
| --- | --- | --- |
| **2013 (6 months)** | 8978 | 12.9 |
| **2014** | 17,630 | 12.0 |
| **2015** | 13,201 | 10.4 |
| **2016** | 12,934 | 9.7 |
| **2017** | 10,827 | 8.8 |

Appendix Table 6. Most frequent primary diagnoses for Secondary-OUD* hospitalizations.

| **Rank** | **ICD-9** | **ICD-10** |
| --- | --- | --- |
| 1 | 2920 Drug Withdrawal (22.5%) | F1020 Alcohol Dependence, Uncomplicated (10.0%) |
| 2 | 30390 Other and Unspecified Alcohol Dependence Unspecified Drunkenness (5.8%) | F332 Major Depressive Disorder, Recurrent Severe Without Psychotic Features (5.9%) |
| 3 | 29633 Major Depressive D/O Recur Episode Sev W/O Psychotic Behavior (4.6%) | F10230 Alcohol Dependence With Withdrawal, Uncomplicated (5.8%) |
| 4 | 29181 Alcohol Withdrawal (3.1%) | F1320 Sedative, Hypnotic Or Anxiolytic Dependence, Uncomplicated (3.9%) |
| 5 | 311 Depressive Disorder Not Elsewhere Classified (3.0%) | F329 Major Depressive Disorder, Single Episode, Unspecified (2.9%) |
| 6 | 30410 Sedative Hypnotic/Anxiolytic Dependence Unspecified (2.6%) | **A419 Sepsis, Unspecified Organism (2.5 %)** |
| 7 | 29690 Unspecified Episodic Mood Disorder (2.3%) | F13230 Sedative, Hypnotic Or Anxiolytic Dependence With Withdrawal, Uncomplicated (1.8%) |
| 8 | 30391 Other & unspecified Alcohol Dependence Cont Drunkenness (2.1%) | F319 Bipolar Disorder, Unspecified (1.5%) |
| 9 | 29620 Major Depressive Disorder Single Episode Unspec (1.7%) | **L03113 Cellulitis Of Right Upper Limb (1.3 %)** |
| 10 | 30480 Comb Drug Depend Exclude Opioid Drug Unspecified Abs (1.4%) | F1520 Other Stimulant Dependence, Uncomplicated (1.2 %) |
| 11 | 29680 Bipolar Disorder Unspecified (1.3 %) | O99320 Drug Use Complicating Pregnancy, Unspecified Trimester (1.0%) |
| 12 | **0389 Unspecified Septicemia (1.3 %)** | F331 Major Depressive Disorder, Recurrent, Moderate (0.8 %) |
| 13 | 29630 Major Depressive Disorder Recurrent Episode Unspecified (1.2%) | F39 Unspecified Mood [affective] Disorder (0.8%) |
| 14 | 29284 Drug-Induced Mood Disorder (1.1%) | F339 Major Depressive Disorder, Recurrent, Unspecified (0.8%) |
| 15 | **6823 Cellulitis And Abscess Of Upper Arm And Forearm (0.9%)** | F3181 Bipolar Ii Disorder (0.8%) |
| 16 | 29650 Bipolar I D/O Most Recent Epis Depressed Uns (0.8%) | J189 Pneumonia, Unspecified Organism (0.7%) |
| 17 | 5770 Acute Pancreatitis (0.7%) | K859 Acute Pancreatitis, Unspecified (0.7%) |
| 18 | 2989 Unspecified Psychosis (0.7%) | N179 Acute Kidney Failure, Unspecified (0.7%) |
| 19 | 29632 Major Depressive Disorder Recurrent Episode Moderate (0.7%) | F314 Bipolar Disorder, Current Episode Depressed, Severe, Without Psychotic Features (0.7 %) |
| 20 | 29623 Major Depressive D/O 1 Epis Severe W/O Mention Psychotic Behavior (0.6%) | F333 Major Depressive Disorder, Recurrent, Severe With Psychotic Symptoms (0.7%) |

*Secondary-OUD hospitalizations are hospitalizations where OUD is not the primary diagnosis. Alcohol-related diagnoses are underlined, infection-related diagnoses are bolded.

Appendix Table 7. Discharge status codes used to indicate discharge to an outpatient setting.

| Discharged to home or self-care |
| --- |
| Discharged/transferred to home health service |
| Left against medical advice |
| Discharged/transferred to home IV drug therapy |
| Discharged/transferred to hospice home |
| Transfer to disaster alternative care site |
| Transfer/referred to other facility for outpatient services |
